# Why "Radiation Oncology"

**DOI:** 10.1186/1748-717X-1-1

**Published:** 2006-02-28

**Authors:** Claus Belka, Kevin A Camphausen

**Affiliations:** 1 Department of Radiation Oncology, University Tübingen, Germany; 2 National Cancer Institute Bethesda, Radiation Oncology Branch, Bethesda, USA

## Abstract

Radiotherapy continues to be a major treatment for solid tumours and is a cornerstone of modern oncology. The term 'radiation oncology' describes the integration of radiation therapy into the complexity of multi-modal therapy. Over the last ten years the crucial role of radiation therapy as part of multi-modality protocols in cancer care has been documented in numerous Phase III trials. Advances in treatment technology as well as the underlying biology of tumour resistance mechanisms will further strengthen the role of radiation oncology. The scientific role of radiation oncology is reflected by the increase in the number of papers related to radiation oncology in resources like Medline. In order to reflect the growing scientific importance of radiation oncology, radiation physics and radiation biology, we have initiated *Radiation Oncology *as the first open access journal in the field. Open access allows for a rapid and transparent publication process together with an unequalled opportunity to reach the widest reader spectrum possible.

## Introduction

It is predicted that in 2006 there will be 1.25 million non-skin cancers diagnosed in North America, and 75% of these patients will receive radiotherapy sometime during the course of their disease. Although there has been much success in terms of local tumour control using radiotherapy there has been less success in terms of an improvement in overall survival in patients receiving only radiotherapy. Future successes using radiotherapy will hinge on the proper integration of clinical radiotherapy, radiation biology and radiation physics into general treatment approaches and the application of these findings in a timely fashion. We are convinced that research in the areas introduced in more detail in the following paragraph will improve the understanding of how radiation acts on tumours and ultimately change the clinical practice.

### Hot topics in radiation oncology

Generally, several distinct hot topic areas may be distinguished. In the field of radiation biology we believe that cell death research aiming to dissect the pathways underlying radiation-induced inactivation of clonogenic tumor cell death is of crucial importance [1]. In addition, many lines of evidence point to the fact that molecular changes introduced by hypoxia and the micromilieu will impact on the efficacy of radiation treatments [2]. Furthermore, radiation sensitivity is controlled by a wide array of growth factors, cytokines, adhesion molecules derived from the tumour stroma or the malignant cell itself. All of these factors are clearly targets for therapeutic intervention [3].

In order to individually tailor a treatment approach it is of crucial importance to define sets of molecules relevant for the prediction of clinical responses. In this regard, it seems likely that complex expression patterns rather than single molecules will guide clinical decisions [4,5]. Since therapeutic gain is the result after balancing tumour cell kill against normal tissue damage, research regarding the molecular and cellular mechanisms of side effects has the potential to positively influence radiation oncology.

Independently from radiation biology, radiation technology in combination with new imaging modalities will strongly improve outcomes. In this regard, the introduction of intensity modulated radiotherapy (IMRT) based on the results of functional imaging studies (PET, MRI) will definitively change our daily radiation practice, although many problems still have to be solved [6]. Although the use of charged particles has high potential impact [7], it remains speculative in how far these modalities will change the daily treatment practice in a near future as the costs of such treatments are extremely high and access to dedicated treatment facilities is limited.

In order to establish a platform for a rapid and virtually unlimited distribution of new results from any of the fields mentioned above we have chosen to initiate the open access journal *Radiation Oncology*.

### Open access

Open access has three broad benefits for science, medicine and the general public.

First, authors are assured that their work is disseminated to the widest possible audience: all articles are archived in several freely accessible archives, such as PubMed Central (USA), the open repositories at the University of Potsdam (Germany), within INIST (France), and within e-Depot (Netherlands).

Second, the information available to readers will not be limited by their library's budget as all articles are freely and universally accessible online at no cost to the reader.

Third, the results of publicly funded research will be accessible to all taxpayers and not just those with proper library access as the authors hold copyright of their work and grant anyone the right to reproduce and disseminate the article.

In addition, the review process, considered to be of crucial importance for scientific quality, is available to the whole scientific community as soon as the paper is published (every paper will be published alongside its prepublication history). Furthermore, for reasons of clarity and fairness the reviewers do not remain anonymous. We are convinced that both features help keep the whole review process as transparent as possible.

### Submission policy

Manuscripts are submitted to *Radiation Oncology *using the online submission system [8]. Submitted manuscripts will be handled by the Editor-in-chief or assigned to a member of our Editorial Board [9], who will seek critical reviews of the manuscript. Once accepted, articles will be published online immediately and will be listed in PubMed soon after.

We look forward to receiving your submissions.
